# A Uniform Concept for Error Estimation in Gamma-Ray Spectrometry

**DOI:** 10.6028/jres.093.125

**Published:** 1988-06-01

**Authors:** Péter Zagyvai, Lajos György Nagy, József Solymosi

**Affiliations:** Technical University of Budapest, Department of Physical Chemistry Hungary

## Introduction

The estimated errors of peak areas are primarily important results for the complete analytical process (e.g., activation analysis or environmental radiocontamination assay). Many excellent program routines were offered in the literature for determining peak areas; however, no unambiguous solution has been given so far for assigning accurate error estimation methods.

As an introduction to our “uniform concept” some basic principles are to be stated:

– The error estimation would establish an accurate confidence limit around the calculated results: this region would include the “true value,” Thus the error is a measure of accuracy rather than that of precision.
– The above condition is met only if the estimated error combines all statistical (random) and systematic (bias) errors.
– The methods for peak area determination and error estimation, respectively, would be closely interdependent.
– The actual formulation is strongly related to the given detection system.

## Theory

Of course it is necessary that a uniform method be used for calculating peak areas of singlets and multiplets as well. The “apportioning” method proved satisfactory for resolving overlaps:

$$I_{i}=\frac{a_{i}\cdot s_{i}}{\sum_{j=1}^{K}a_{j}\cdot s_{j}}\cdot I_{\text{ST}}$$ (1)

where

*I*_ST_: is the total count rate of the multiplet determined by summing and baseline subtraction,
*K*: is the number of overlapping peaks
*I*_i_: is the count rate of the i’th peak,
*a*: is the net peak height, and
*s*: is the peak width.

Peak heights are computed from least squares fit with fixed and previously calibrated widths. The error of the i’th count rate is given by eq (2):

$${\left(\Delta I_{i}\right)}^{2}=\frac{{\left(\Delta a_{i}\right)}^{2}}{a_{i}^{2}}+\frac{\sum_{j=1}^{K}{\left(\Delta a_{j}\right)}^{2}}{{\left(\sum_{j=1}^{K}a_{j}\right)}^{2}}+\frac{{\left(\Delta I_{\text{ST}}\right)}^{2}}{I_{\text{ST}}^{2}}\cdot I_{i}^{2}$$ (2)

The first two terms represent the “bias” error, the third one contains the “random” error. Peak height errors Δ*a* are generated in the least squares fit.

The count rate *I* for singlets is the maximum of the summed and baseline-subtracted net counts (*I*_S_) and the integral of the fitted peak shape function (*I*_F_) If we consider this step as a “one component apportioning,” the error of the count rate is given by eq (3):

$${\left(\Delta I\right)}^{2}=\frac{{\left(\Delta a\right)}^{2}}{a^{2}}+\frac{{\left(\Delta a\right)}^{2}}{a^{2}}+\frac{{\left(\Delta I_{S}\right)}^{2}}{I_{S}^{2}}\cdot I^{2}$$ (3)

The resemblance of eqs (2) and (3) is quite clear.

If the radioactivities attributed to the identified isotopes are calculated not individually but in a common least squares procedure (called interference correction), the estimated peak area errors must be applied as weight factors in the least squares solution.

## Results

Numerous reference materials were analyzed to confirm the reliability of both the peak area computation and error estimation methods (such as Bowen’s kale, NBS and IAEA reference materials). In testing the experimental standard deviations, it was revealed that some analytical results were biased. However, most results were still reliable because the known true values in most cases did fall into the calculated confidence intervals.

## Summary

We elaborated a uniform formulation for calculating estimated count rate errors in gamma spectrometry. Experimental results confirmed that our method gave accurate confidence limits combining both random and bias errors.

